# Association between oxidative balance score and systemic immune-inflammation index: NHANES 2003–2018

**DOI:** 10.1371/journal.pone.0329540

**Published:** 2025-08-01

**Authors:** Yongling Niu, Ziyi Fan, Zelin Wang, Shufen Liang

**Affiliations:** 1 Department of Neurology, The Second Hospital of Shanxi Medical University, Taiyuan, China; 2 Department of Oncology, Third Hospital of Shanxi Medical University, Shanxi Bethune Hospital, Shanxi Academy of Medical Sciences, Tongji Shanxi Hospital, Taiyuan, China; 3 Department of Laboratory, The Second Hospital of Shanxi Medical University, Taiyuan, China; University of Brasilia: Universidade de Brasilia, BRAZIL

## Abstract

**Background:**

As we all known oxidative stress and inflammation were interdependent and interconnected. Oxidative balance score (OBS) could assess the antioxidant capacity of an individual’s body. The purpose of this study sought to investigate the connection between OBS and the recently proposed inflammatory indicator——Systemic immune-inflammation index (SII).

**Methods:**

The study incorporated 16,080 participants from National Health and Nutrition Examination Survey (NHANES) database (2003–2018). We utilized weight multivariable linear regression analyses to assess this association. Additionally, subgroup analyses and linear relationships were performed for deeper insights. The robustness of the findings was ensured by conducting multiple sensitivity analyses.

**Results:**

In the multivariate model using the first OBS category as the reference, higher OBS quartiles had a significantly negative association with SII [β = −51.52 (95%CI: −68.31, −34.73), *P* for trend < 0.001]. The interaction test showed that age (*P* < 0.001) and hypertension (*P* = 0.043) had a significant effect on this connection. Restricted cubic splines plot with four knots illustrated a linear relationship between OBS and SII (*P*-non-liner = 0.677). The findings from the sensitivity analysis aligned with the outcomes of the primary analysis, confirming the consistency and reliability of the results.

**Conclusions:**

OBS based on diet and lifestyle had a strongly negative association with SII. This study emphasized the importance of improving an individual’s overall antioxidant status through diet and lifestyle changes, highlighting its effectiveness in reducing inflammation index.

## Introduction

Oxidative stress was a disorder of antioxidant and prooxidants system. Reactive oxygen species (ROS), one prooxidant mainly produced by mitochondria, affected many crucial signaling pathways and caused DNA damage, lipid peroxidation, and damage to proteins and enzymes [1,2]. Published papers indicated that the initiation and progression of several diseases were already inseparable from oxidative stress, for instance, cancer, cardiovascular disease, and neurological disorders, among others [3]. There was substantial evidence indicating that the relationship between oxidative stress and inflammation was interdependent and interconnected. Oxidative stress could promote the synthesis and release of inflammatory cytokines, and inflammatory cells releasing lots of ROS simultaneously could exacerbate oxidative stress [4,5]. ROS have been reported to activate the AP-1 pathway and then expression of pro-inflammatory genes [6]. Therefore, it was very imperative to explore the occurrence and treatment of diseases based on both these aspects in the study.

Oxidative Balance Score (OBS) is designed to assess a person’s total antioxidant capacity [7]. A separate factor’s impact on the complete oxidative balance system was relatively minimal [8]. Hence, this score combined 20 antioxidant and prooxidant compositions from dietary and lifestyle factors, typically assigning positive points to antioxidant components [9]. Generally, higher OBS signifies that an individual has strong antioxidant capacity. Several studies have revealed a strong inverse relationship between OBS and the risk of depression was demonstrated [8,10], and a decrease in OBS was linked to a higher risk of cardiovascular disease, diabetes, kidney disease, and so on [11–13]. The Systemic immune-inflammation index (Sll), which could evaluate systemic inflammatory response, was an integrated and innovative indicator derived from lymphocyte, neutrophil, and platelet counts [14]. It was originally created by Hu et al. to evaluate the prognosis of individuals with hepatocellular carcinoma [15], as well as the prognosis of gastroesophageal adenocarcinoma [16]. In addition, some studies have also observed that SII could precisely assess inflammation status. For instance, SII may predict rheumatoid arthritis risk in US adults [14] and shows a positive correlation with higher urinary albumin excretion [17]. Systemic Inflammatory Response Index (SIRI), a novel composite index, integrates three independent white blood cell subsets. SIRI, like SII, can indicate the level of inflammation in the body.

OBS and SII were both novel indicators proposed in recent years regarding overall oxidative balance and inflammation. Although previous studies have found that oxidative stress and inflammation were interrelated, this connection requires further validation based on large population surveys. To perfect this knowledge gap, this study used National Health and Nutrition Examination Survey (NHANES) data to explor the relationship between OBS and SII in US adults. To date, researchers have yet to employ the NHANES data to investigate the problem.

## Methods

### Study population

The study’s data encompassed eight cycles of subjects (2003–2018) drawn from the NHANES, a broad cross-sectional investigation designed to survey the whole nation’s health and nutritional levels in the US, conducted biennially. NHANES employed a sophisticated multistage probability sampling method and incorporated interviews, physical assessments, and laboratory data. Data from the NHANES were freely accessible at https://www.cdc.gov/nchs/nhanes/, and participants provided informed consent before participation in the NHANES study. (Website access time: December 15, 2023).

**Fig 1:**
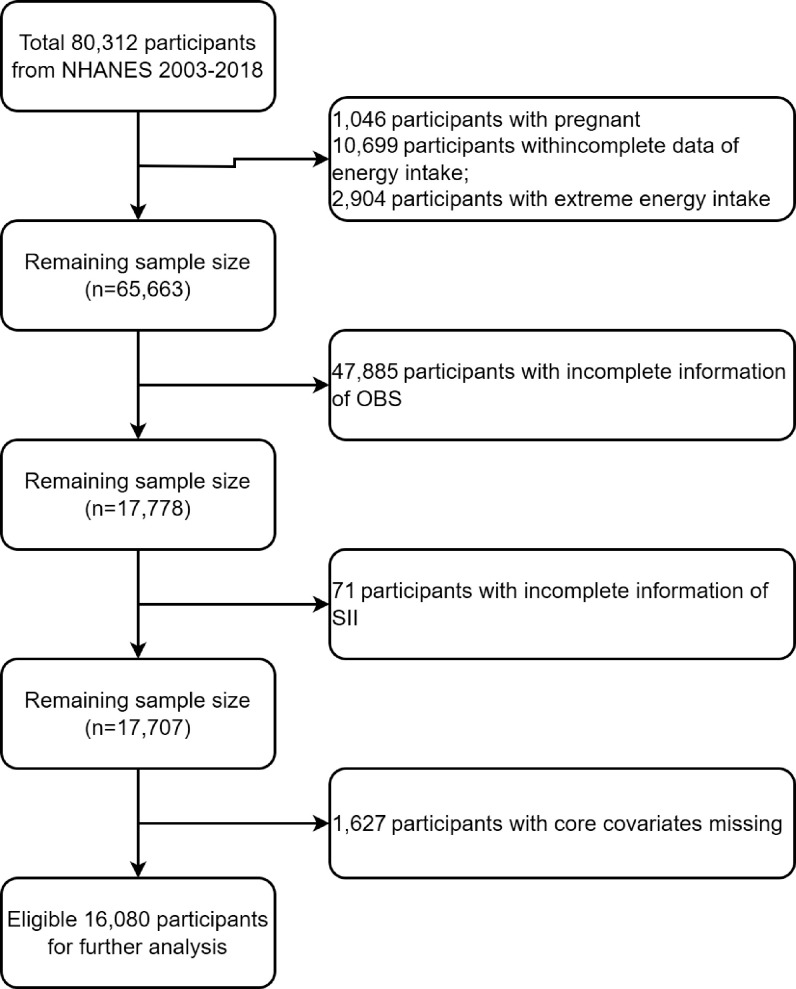
Flowchart portraying the sample selection.

Among 80,312 participants from eight survey cycles of NHANES 2003–2018, our study excluded pregnant (n = 1,046), incomplete data of energy intake (n = 10,699) and extreme energy intake participants (men consuming < 800 kcal/day or > 4200 kcal/day, and women consuming < 500 kcal/day or > 3500 kcal/day) (n = 2,904, total n = 14,649), and participants without complete information about OBS, SII, core covariates (Fig 1). Finally, 16,080 participants were incorporated for further analysis.

### Oxidative balance scores

According to previous studies, most of the calculations about participants’ OBS selected 16 dietary and 4 lifestyle components to map to the body’s oxidative stress levels (9). The composition of OBS include five pro-oxidants (Total fat, Iron, Alcohol, Body mass index, Cotinine) and fifteen antioxidants (Dietary fiber, Carotene, Riboflavin, Niacin, Calcium, Total Folate, Vitamin B6, Vitamin B12, Vitamin C, Vitamin E, Magnesium, Copper, Zinc, Selenium). Dietary intake was acquired through a review of total nutrient intake during the interview in this study. Notably, the primary metabolite of nicotine was cotinine, which could be used as a marker of smoking. All continuous variables of OBS components were divided into tertile on a weighted basis based on their distribution. For antioxidants, participants from tertile 1 through tertile 3 received scores ranging from 0 to 2, while the scoring was inverted for pro-oxidants. Furthermore, alcohol scores were allocated based on gender-specific alcohol consumption rates: less than 12 alcoholic beverages in the past year scored 2; less than 1 drink daily for women or less than 2 drinks daily for men scored 1; and all other participants scored 0(10). To calculate physical activity, we multiply the metabolic equivalent (MET) score by the frequency of each physical activity per week and the duration of each physical activity (S1 Table) [18]. Ultimately, the sum of the scores at each composition is OBS. For the convenience of research, we transformed the continuous variable OBS into quartiles and tertiles variable [8].

### Systemic immune-inflammatory index

The definition of SII was determined by prior research information, wherein the calculation formula entails multiplying the platelet count by the neutrophil count and then dividing by the lymphocyte count [14,15]. The calculation formula of SIRI is monocyte counts multiplied by the neutrophil count and then divided by the lymphocyte count. The complete blood count (CBC) was conducted using the Beckman Coulter DxH 800 instrument with SP’s EDTA blood tubes by the NHANES mobile examination center (MEC).

### Covariates

In our current study, the covariates may affect the SII include age (continuous variable or 20–40, 40–60, more than 60), gender (male; female), education level (below high school; high school or above), ethnicity (Mexican American; Non-Hispanic White; Non-Hispanic Black; Other), poverty income ratio (PIR) (less than 1; 1–3; more than 3), marital status (married/cohabiting; never married; widowed/divorced/separated), diabetes (yes; no), hypertension (yes; no) [8]. Specifically, hypertension was assessed by blood pressure measurement, self-reported history of hypertension, or taking antihypertensive drugs [19]. Diabetes was assessed by fasting glucose, glycohemoglobin, self-reported history of diabetes, or taking diabetic pills [20].

### Statistical analyses

All analyses used the weights derived from the 24-h dietary recall interview to adjust for the complex multistage probability sampling design of NHANES in this study (Formula for weight calculation: 1/8*WTDRD1. Participant baseline characteristics were described by means with standard deviations (SD) for continuous variables, the number of cases (n) with percentages (%) for categorical variables. Multicollinearity was assessed using the variance inflation factor. However, no significant multicollinearity (VIF > 2) was observed among the variables in this study (S2 Table). We analyzed the association of the OBS quartile and SII by constructing three multivariate linear regression models. Mode 1 was unadjusted for covariates. Model 2 was adjusted for age, gender, and ethnicity. Model 3 further incorporated adjustments for education level, family poverty income, marital status, diabetes, and hypertension. Moreover, subgroup analyses were conducted by age (20–40, 40–60, > 60), gender, ethnicity, education level, family poverty income, marital status, diabetes, and hypertension, and we conducted tests for multiplicative interactions by using the Wald test. Finally, a restricted cubic spline plot with four knots was employed to investigate the potential nonlinear relationship. To ensure the robustness of the result, we additionally conducted three sensitivity analyses, including 1) the OBS was converted into categorical variables (tristile) for repeated analysis; 2) in order to prevent bias in the analysis of the continuous variable SII, the main analysis was repeated based on SII tristile transformation [21]; 3) the relationship between OBS and SII was reanalyzed without adjusting for weight. Considering that SII were right-skewed distributed, SII was ln-transformed when converting into categorical variables. Statistical analyses were carried out using R software (version 4.2.2), considering a *P*-value < 0.05 as statistically significant.

## Results

### Baseline characteristics of the study population

Among the 16,080 participants, the majority of participants were about 45 years old, non-Hispanic White ethnicity (non-weighted frequency, 50.32%), married/cohabiting (non-weighted frequency, 61.52%), with higher education levels (non-weighted frequency, 83.88%). Table 1 depicts a breakdown of the baseline characteristics of the participants across the OBS quartile. In addition, we found that higher OBS exhibited a higher likelihood of being female (weighted frequency, 73.6%), and males conversely showed a lower OBS (weighted frequency, 66.3%) (*P* < 0.001). It was important to note that SII gradually decreased as OBS increased (*P* < 0.001). The study’s findings represent a weighted population of 304,004,766 people.

**Table 1:** Baseline characteristics of study population.

Characteristics	Q1	Q2	Q3	Q4	*P-value*
N	4401	3528	4139	4012	
Age (mean (SD))	43.43 (15.85)	45.04 (15.88)	45.27 (15.72)	45.06 (15.34)	*<0.001*
**Gender (%)**					*<0.001*
Female	2716 (66.3)	1787 (53.6)	1692 (44.5)	1027 (26.4)	
Male	1685 (33.7)	1741 (46.4)	2447 (55.5)	2985 (73.6)	
**Race (%)**					*<0.001*
Mexican American	588 (7.0)	467 (6.4)	614 (7.1)	604 (7.4)	
Non-Hispanic Black	1153 (13.6)	667 (8.9)	640 (6.8)	475 (5.0)	
Non-Hispanic White	1981 (69.0)	1768 (74.3)	2166 (75.9)	2177 (77.1)	
Other	679 (10.4)	626 (10.4)	719 (10.2)	756 (10.4)	
**Marital status (%)**					*<0.001*
Married/cohabiting	2360 (57.8)	2124 (64.5)	2697 (68.0)	2715 (70.8)	
Never married	1013 (21.7)	724 (18.7)	768 (17.7)	773 (18.2)	
Widowed/divorced/separated	1028 (20.5)	680 (16.8)	674 (14.3)	524 (11.0)	
**Education level (%)**					*<0.001*
Below high school	862 (12.9)	608 (11.0)	619 (9.1)	503 (7.4)	
High School or above	3539 (87.1)	2920 (89.0)	3520 (90.9)	3509 (92.6)	
**PIR (%)**					*<0.001*
<1	996 (16.3)	551 (9.9)	594 (8.6)	482 (7.5)	
>3	1572 (47.4)	1562 (56.5)	2055 (61.9)	2184 (66.0)	
1-3	1833 (36.3)	1415 (33.6)	1490 (29.4)	1346 (26.5)	
**Diabetes (%)**					0.262
No	3826 (90.4)	3094 (90.7)	3657 (91.4)	3603 (91.9)	
Yes	575 (9.6)	434 (9.3)	482 (8.6)	409 (8.1)	
**Hypertension (%)**					0.215
No	2640 (65.2)	2174 (65.1)	2578 (65.7)	2636 (67.8)	
Yes	1761 (34.8)	1354 (34.9)	1561 (34.3)	1376 (32.2)	
SII (mean (SD))	567.22 (329.94)	545.01 (311.53)	536.04 (307.46)	502.29 (268.83)	*<0.001*

N: Number of participants; PIR: poverty income ratio; SII: Systemic immune-inflammation index; SD: Standard deviation; (%): The proportion of categorical variables.

### Association between OBS and SII/SIRI

Table 2 presents the assessment of the association between OBS quartiles and SII using weighted linear regression. In Model 1, higher OBS quartiles were significantly linked with a greater decrease in SII when using the first OBS category as a reference [β = −64.93 (95%CI: −80.95, −48.91), *P* for trend < 0.001]. Even after adjusting for potential confounding variables, all correlations were still statistically significant [β = −61.14 (95%CI: −78.21, −44.06)], and *P* for trend < 0.001. In the fully adjusted model (Model 3), the SII decreased by 51.52 for each unit, which increased in OBS when the OBS was the highest quartile (compared to quartile 1). Moreover, 3 sensitivity analyses consistently validated the reliability of the results, which included repeated analysis with OBS tristile (S3 Table), conversion into categorical variables with SII (S4 Table), and exclusion of the use of complex survey design (S5 Table).

**Table 2:** Survey-weighted association between OBS and SII.

	Model 1		Model 2		Model 3	
	Beta Estimate (95% CI)	*P*-value	Beta Estimate (95% CI)	*P*-value	Beta Estimate (95% CI)	*P-*value
Q1	Ref	/	Ref	/	Ref	/
Q2	−22.21 (−39.84, −4.59)	*0.014*	−23.77 (−42.06, −5.48)	*0.011*	−19.89 (−37.77, −2.01)	*0.030*
Q3	−31.17 (−46.48, −15.87)	*<0.001*	−31.97 (−47.89, −16.05)	*<0.001*	−25.43 (−41.46, −9.40)	*0.002*
Q4	−64.93 (−80.95, −48.91)	*<0.001*	−61.14 (−78.21, −44.06)	*<0.001*	−51.52 (−68.31, −34.73)	*<0.001*
*P* for trend	/	*<0.001*	/	*<0.001*	/	*<0.001*

Model 1: did not adjust covariates. Model 2: Further adjustment for age, gender, and ethnicity. Model 3: Further adjustment for education level, family poverty income, marital status, diabetes, and hypertension. CI: confidence intervals; Ref: reference.

S6 Table presents the assessment of the association between OBS quartiles and SIRI. In Model 1, higher OBS quartiles were significantly linked with a greater decrease in SIRI when using the first OBS category as a reference [β = −0.05 (−0.09, −0.01), *P* for trend = 0.038]. Even after adjusting for potential confounding variables, all correlations were still statistically significant [β = −0.16 (−0.21, −0.12)], and *P* for trend < 0.001. In the fully adjusted model (Model 3), the SIRI decreased by 0.13 for each unit, which increased in OBS when the OBS was the highest quartile (compared to quartile 1).

### Subgroup analysis and interaction test

**Fig 2:**
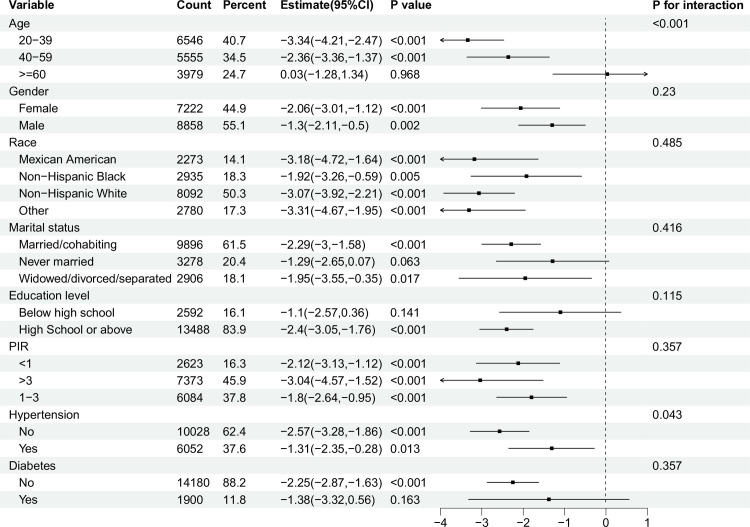
Forest map of subgroup analysis between OBS and SII. OBS: Oxidative Balance Score; SII: Systemic immune-inflammation index; PIR: poverty income ratio; CI: confidence intervals.

Through conducting subgroup analyses, we determined that some characteristics did not consistently correlate with reduced SII levels. This allowed us to assess the impact of these factors on different groups (Fig 2). For age (>=60) [β = 0.03(95%CI: −1.28,1.34)], marital status (Never married) [β = −1.29(95%CI: −2.65,0.07)], education level of below high school [β = −1.1(95%CI: −2.57,0.36)], and diabetes [β = −1.38(95%CI: −3.32,0.56)], this correlation lacked statistical significance (*P* > 0.05). In addition, when analyzing subgroups based on gender, race, PIR, and hypertension, significant negative associations between OBS and SII were observed. In particular, the interaction test showed that age and hypertension had a significant effect on this connection (*P* < 0.05).

### Nonlinear relationship analyses

**Fig 3:**
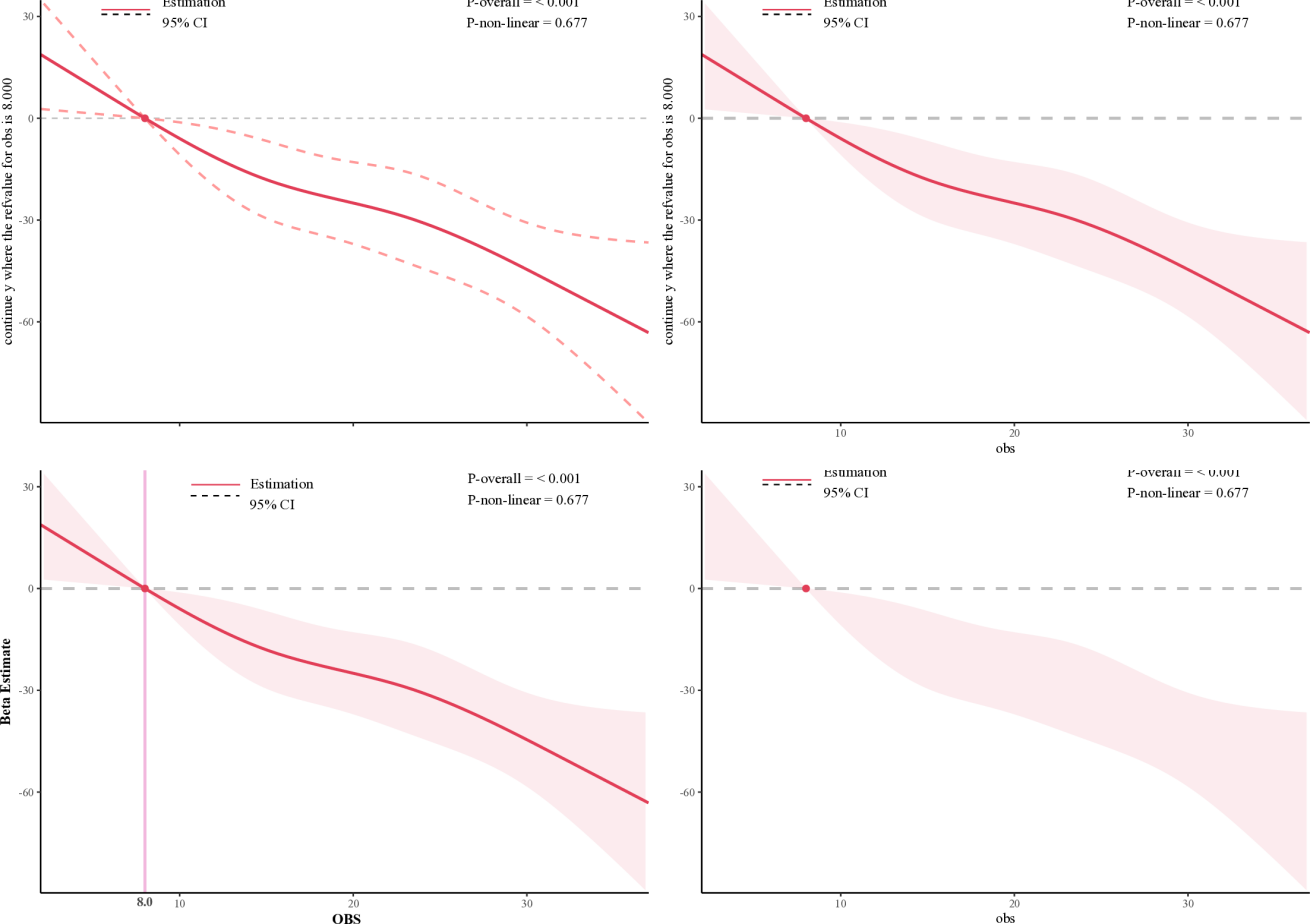
Restricted cubic spline plot between OBS and SII. The shaded areas represent the 95% CI (Adjusted for age, gender, ethnicity, education level, family poverty income, marital status, diabetes, and hypertension) OBS: Oxidative Balance Score; SII: Systemic immune-inflammation index; CI: confidence intervals.

Adjusting for all covariates, we detected a significant linear relationship between OBS and SII in a restricted cubic splines plot with four knots (*P*-non-linear = 0.677) (Fig 3). The result presents a negative relationship between OBS and SII, indicating that the SII decreased with the OBS increase. Moreover, the threshold point of SII was determined to be 8.0, which was an OBS reference value when beta equaled 0. When the SII was less than the threshold point, the beta estimate was greater than 0, and vice versa.

## Discussion

This study ultimately encompassed 16,080 participants from eight cycles of NHANES subjects for cross-sectional analysis, including 8,858 males and 7,222 females. In general, our results illustrated a negative association between OBS and SII among adults, indicating that higher OBS scores were associated with lower SII in participants. Further, subsequent adjustment for all covariates revealed that the relationship was linear by a restricted cubic splines plot with four knots. Notably, there was an interaction of age and hypertension between OBS and SII. Additionally, three sensitivity analyses also further corroborated the robust stability of the association between OBS and SII. Our results align with prior research suggesting that adherence to a rich antioxidant diet and lifestyle played a crucial role in relieving inflammation.

To our knowledge, this is the inaugural investigation examining the correlation between OBS and SII using NHANES data. Oxidative stress and inflammation are widely recognized to play crucial roles in the onset of numerous diseases. Unstable and highly reactive ROS must be kept at a low level in order to maintain cellular homeostasis and function [22]. However, there was specific evidence that the high level of ROS produced by NADPH oxidases was found in chondrocytes isolated from osteoarthritis cartilage [23,24]. Moreover, ROS regulated endothelial cell function and vascular remodeling of hypertension from cumulative evidence [25]. ROS also stimulated the PI3K/Akt-MAPK pathway, which inhibited the expression and activity of NOS, thereby reducing NO, which was an important signaling molecule in maintaining vascular homeostasis [26]. It is worth noting that the occurrence of cancer is also inseparable from the involvement of oxidative stress and inflammation. During chronic inflammation, various immune cells that gathered in damaged areas led to increased release and accumulation of ROS at this site [27]. Next, ROS could directly initiate carcinogenesis or mediate related signaling pathways. Superoxide anion (O_2_^-^), H_2_O_2_, and NO all played important roles in the development of cancer [28]. Meanwhile, epidemiological studies have consistently revealed a connection between oxidative stress and inflammation. For instance, Qu et al. demonstrated that individuals in the highest quartile of OBS experienced a 29% reduction in the risk of periodontitis [29]. Wang et al. further elucidated that diets could mitigate oxidative stress-mediated biological aging [30]. Extensive clinical evidence supports the notion that oxidative stress and inflammation serve as important causes of numerous diseases, including atrial fibrillation [31], accelerated aging [32], depression [33], and more.

The OBS used in this study involved diet and lifestyle to represent the state of oxidative balance in the internal environment. Although the link of diet or lifestyle to inflammatory states has been proven, there is not much discussion of the two together. Tripathi et al. found that dietary supplementation with vitamin B12 and folate was effective in the prevention and treatment of non-alcoholic steatohepatitis [34]. Vitamin C is widely recognized as a crucial circulating antioxidant with anti-inflammatory properties. Evidence suggests that early administration of high-dose intravenous vitamin C may help mitigate the production of excessive ROS and improve outcomes in ischemia/reperfusion injury [35]. Notably, recent studies on diet and inflammation have focused on an individual’s gut microbiota status, which can be modulated by diet. It was a key mediator of vitamin absorption because it produces human nonproductive vitamins [36]. Vangay et al. found that many US immigrant populations developed metabolic diseases associated with missing gut microbiome diversity and function [37]. In an experiment where African Americans and rural Africans swapped diets for two weeks, the food changes resulted in a remarkable change in microbiota known to affect cancer risk [38].

According to the report, regular physical activity was an important part of the reduction in inflammatory parameters and oxidative stress biomarkers. Physical exercise was considered an inhibitory effect of inflammation and microglial activation in Alzheimer’s disease, Parkinson’s disease [39]. In addition, chronic smoking and drinking promoted significant increase in peripheral cytokine concentrations, especially pro-inflammatory cytokines [40,41]. Obesity is becoming an epidemic worldwide and increases the risk for many diseases, including type 2 diabetes, cardiovascular disease, and so on. It has been recognized that obese patients had easily increased accumulation and inflammatory polarization of immune cells, leading to obesity-linked metabolic dysfunctions [42].

Our research had several advantages. First, we used nationally representative population surveys based on NHANES data and selected a weight multistage regression analysis to ensure high data quality. Second, the OBS as an overall indicator, including diet and lifestyle, could better and more comprehensively comprehend the relationship between an individual’s overall antioxidant status and inflammation. Third, the accuracy of the results is ensured by adjusting for several confounding factors. Finally, multiple sensitivity analyses ensured the robustness of the finding.

Despite this, there are several limitations to be aware of. First, as this study adopted a cross-sectional design, no causal correlations can be inferred. Second, Assessing the oxidation balance of the organism is complex, so more indicators need to be added to improve the accuracy of the results. In addition, adjusting for certain variables may not have fully accounted for residual and unmeasured confounders, potentially introducing bias into the analysis. Therefore, further investigations are needed to perfect the result.

## Conclusions

In conclusion, this study, utilizing a nationally representative population, indicates a linear negative correlation between OBS and SII. The result showed that age and hypertension had an interaction in these associations. This study emphasized the importance of promoting an individual’s overall antioxidant status through diet and lifestyle, which may aid in reducing inflammation levels. Moving forward, further research is needed to delve into the precise mechanisms underlying oxidative stress and inflammation.

## Supporting information

S1 Table Scoring rubric of OBS. (XLSX)

S2 Table Multicollinearity of variables. (XLSX)

S3 Table Survey-weighted association between OBS tertile and SII. (XLSX)

S4 Table Survey-weighted association between OBS and SII tertile. (XLSX)

S5 Table Not weight association between OBS and SII. (XLSX)

S6 Table Survey-weighted association between OBS4 and SIRI. (XLSX)

## Abbreviations

OBS: oxidative balance score
SII: systemic immune-inflammation index
NHANES: National Health and Nutrition Examination Survey
ROS: reactive oxygen species
MET: metabolic equivalent
CBC: complete blood count
MEC: mobile examination center
SD: standard deviations
IL-1β: interleukin-1β
TNFα: tumor necrosis factor α

## References

[1] Franczyk B, Gluba-Brzózka A, Rysz-Górzyńska M, Rysz J. The role of inflammation and oxidative stress in rheumatic heart disease. Int J Mol Sci. 2022;23(24):15812. doi: 10.3390/ijms232415812 36555452 PMC9781220

[2] Juan CA, Pérez de la Lastra JM, Plou FJ, Pérez-Lebeña E. The chemistry of reactive oxygen species (ROS) revisited: outlining their role in biological macromolecules (DNA, Lipids and Proteins) and induced pathologies. Int J Mol Sci. 2021;22(9):4642. doi: 10.3390/ijms22094642 33924958 PMC8125527

[3] Pizzino G, Irrera N, Cucinotta M, Pallio G, Mannino F, Arcoraci V, et al. Oxidative stress: harms and benefits for human health. Oxid Med Cell Longev. 2017;2017:8416763. doi: 10.1155/2017/8416763 28819546 PMC5551541

[4] Saucedo R, Ortega-Camarillo C, Ferreira-Hermosillo A, Díaz-Velázquez MF, Meixueiro-Calderón C, Valencia-Ortega J. Role of oxidative stress and inflammation in gestational diabetes mellitus. Antioxidants. 2023;12(10). 10.3390/antiox12101812 PMC10604289 37891891

[5] Elmarakby AA, Sullivan JC. Relationship between oxidative stress and inflammatory cytokines in diabetic nephropathy. Cardiovasc Ther. 2012;30(1):49–59. doi: 10.1111/j.1755-5922.2010.00218.x 20718759

[6] Chi P-L, Chen Y-W, Hsiao L-D, Chen Y-L, Yang C-M. Heme oxygenase 1 attenuates interleukin-1β-induced cytosolic phospholipase A2 expression via a decrease in NADPH oxidase/reactive oxygen species/activator protein 1 activation in rheumatoid arthritis synovial fibroblasts. Arthritis Rheum. 2012;64(7):2114–25. doi: 10.1002/art.34371 22231145

[7] Hernández-Ruiz Á, García-Villanova B, Guerra-Hernández EJ, Carrión-García CJ, Amiano P, Sánchez MJ. Oxidative balance scores (OBSs) integrating nutrient, food and lifestyle dimensions: development of the NutrientL-OBS and FoodL-OBS. Antioxidants. 2022;11(2). 10.3390/antiox11020300 PMC8868253 35204183

[8] Liu X, Liu X, Wang Y, Zeng B, Zhu B, Dai F. Association between depression and oxidative balance score: National Health and Nutrition Examination Survey (NHANES) 2005-2018. J Affect Disord. 2023;337:57–65. 37244542 10.1016/j.jad.2023.05.071

[9] Zhang W, Peng S-F, Chen L, Chen H-M, Cheng X-E, Tang Y-H. Association between the oxidative balance score and telomere length from the national health and nutrition examination survey 1999-2002. Oxid Med Cell Longev. 2022;2022:1345071. doi: 10.1155/2022/1345071 35186180 PMC8850082

[10] Li H, Song L, Cen M, Fu X, Gao X, Zuo Q, et al. Oxidative balance scores and depressive symptoms: mediating effects of oxidative stress and inflammatory factors. J Affect Disord. 2023;334:205–12. doi: 10.1016/j.jad.2023.04.134 37149058

[11] Kwon Y-J, Park H-M, Lee J-H. Inverse association between oxidative balance score and incident Type 2 diabetes mellitus. Nutrients. 2023;15(11):2497. doi: 10.3390/nu15112497 37299460 PMC10255164

[12] Son DH, Lee HS, Seol SY, Lee YJ, Lee JH. Association between the oxidative balance score and incident chronic kidney disease in adults. Antioxidants (Basel, Switzerland). 2023;12(2). 10.3390/antiox12020335 PMC9952833 36829895

[13] Chen X, Wang C, Dong Z, Luo H, Ye C, Li L, et al. Interplay of sleep patterns and oxidative balance score on total cardiovascular disease risk: insights from the national health and nutrition examination survey 2005-2018. J Glob Health. 2023;13:04170. doi: 10.7189/jogh.13.04170 38085249 PMC10715456

[14] Liu B, Wang J, Li Y-Y, Li K-P, Zhang Q. The association between systemic immune-inflammation index and rheumatoid arthritis: evidence from NHANES 1999-2018. Arthritis Res Ther. 2023;25(1):34. doi: 10.1186/s13075-023-03018-6 36871051 PMC9985219

[15] Hu B, Yang X-R, Xu Y, Sun Y-F, Sun C, Guo W, et al. Systemic immune-inflammation index predicts prognosis of patients after curative resection for hepatocellular carcinoma. Clin Cancer Res. 2014;20(23):6212–22. doi: 10.1158/1078-0432.CCR-14-0442 25271081

[16] Jomrich G, Paireder M, Kristo I, Baierl A, Ilhan-Mutlu A, Preusser M, et al. High systemic immune-inflammation index is an adverse prognostic factor for patients with gastroesophageal adenocarcinoma. Ann Surg. 2021;273(3):532–41. doi: 10.1097/SLA.0000000000003370 31425286

[17] Qin Z, Li H, Wang L, Geng J, Yang Q, Su B, et al. Systemic immune-inflammation index is associated with increased urinary albumin excretion: a population-based study. Front Immunol. 2022;13:863640. 35386695 10.3389/fimmu.2022.863640 PMC8977553

[18] Tian X, Xue B, Wang B, Lei R, Shan X, Niu J. Physical activity reduces the role of blood cadmium on depression: a cross-sectional analysis with NHANES data. Environ Pollut. 2022;304:119211. 35341822 10.1016/j.envpol.2022.119211

[19] Miao H, Liu Y, Tsai TC, Schwartz J, Ji JS. Association between blood lead level and uncontrolled hypertension in the US Population (NHANES 1999-2016). J Am Heart Assoc. 2020;9(13):e015533. doi: 10.1161/JAHA.119.015533 32573312 PMC7670543

[20] Dong G, Gan M, Xu S, Xie Y, Zhou M, Wu L. The neutrophil-lymphocyte ratio as a risk factor for all-cause and cardiovascular mortality among individuals with diabetes: evidence from the NHANES 2003-2016. Cardiovasc Diabetol. 2023;22(1):267. doi: 10.1186/s12933-023-01998-y 37775767 PMC10541705

[21] Shi L, Zhang L, Zhang D, Chen Z. Association between systemic immune-inflammation index and low muscle mass in US adults: a cross-sectional study. BMC Public Health. 2023;23(1):1416. doi: 10.1186/s12889-023-16338-8 37488531 PMC10367418

[22] Trachootham D, Lu W, Ogasawara MA, Nilsa R-DV, Huang P. Redox regulation of cell survival. Antioxid Redox Sig. 2008;10(8):1343–74. doi: 10.1089/ars.2007.1957 18522489 PMC2932530

[23] Altay MA, Ertürk C, Bilge A, Yaptı M, Levent A, Aksoy N. Evaluation of prolidase activity and oxidative status in patients with knee osteoarthritis: relationships with radiographic severity and clinical parameters. Rheumatol Int. 2015;35(10):1725–31. doi: 10.1007/s00296-015-3290-5 25994092

[24] Lismont C, Nordgren M, Van Veldhoven PP, Fransen M. Redox interplay between mitochondria and peroxisomes. Front Cell Dev Biol. 2015;3:35. doi: 10.3389/fcell.2015.00035 26075204 PMC4444963

[25] Montezano AC, Touyz RM. Oxidative stress, Noxs, and hypertension: experimental evidence and clinical controversies. Ann Med. 2012;44 Suppl 1:S2-16. doi: 10.3109/07853890.2011.653393 22713144

[26] Zhang Z, Zhao L, Zhou X, Meng X, Zhou X. Role of inflammation, immunity, and oxidative stress in hypertension: new insights and potential therapeutic targets. Front Immunol. 2023;13:1098725. doi: 10.3389/fimmu.2022.1098725 36703963 PMC9871625

[27] Hussain SP, Hofseth LJ, Harris CC. Radical causes of cancer. Nat Rev Cancer. 2003;3(4):276–85. doi: 10.1038/nrc1046 12671666

[28] Reuter S, Gupta SC, Chaturvedi MM, Aggarwal BB. Oxidative stress, inflammation, and cancer: how are they linked?. Free Radic Biol Med. 2010;49(11):1603–16. doi: 10.1016/j.freeradbiomed.2010.09.006 20840865 PMC2990475

[29] Qu H. The association between oxidative balance score and periodontitis in adults: a population-based study. Front Nutri. 2023;10:1138488. 10.3389/fnut.2023.1138488 PMC10178495 37187879

[30] Wang X, Sarker SK, Cheng L, Dang K, Hu J, Pan S. Association of dietary inflammatory potential, dietary oxidative balance score and biological aging. Clin Nutri. 2024;43(1):1–10. 10.1016/j.clnu.2023.11.007 37992632

[31] Karam BS, Chavez-Moreno A, Koh W, Akar JG, Akar FG. Oxidative stress and inflammation as central mediators of atrial fibrillation in obesity and diabetes. Cardiovasc Diabetol. 2017;16(1):120. doi: 10.1186/s12933-017-0604-9 28962617 PMC5622555

[32] Papaconstantinou J. The role of signaling pathways of inflammation and oxidative stress in development of senescence and aging phenotypes in cardiovascular disease. Cells. 2019;8(11):1383. doi: 10.3390/cells8111383 31689891 PMC6912541

[33] Bhatt S, Nagappa AN, Patil CR. Role of oxidative stress in depression. Drug Discov Today. 2020;25(7):1270–6. doi: 10.1016/j.drudis.2020.05.001 32404275

[34] Tripathi M, Singh BK, Zhou J, Tikno K, Widjaja A, Sandireddy R, et al. Vitamin B12 and folate decrease inflammation and fibrosis in NASH by preventing syntaxin 17 homocysteinylation. J Hepatol. 2022;77(5):1246–55. doi: 10.1016/j.jhep.2022.06.033 35820507

[35] Spoelstra-de Man AME, Elbers PWG, Oudemans-van Straaten HM. Making sense of early high-dose intravenous vitamin C in ischemia/reperfusion injury. Crit Care. 2018;22(1):70. doi: 10.1186/s13054-018-1996-y 29558975 PMC5861638

[36] Stacchiotti V, Rezzi S, Eggersdorfer M, Galli F. Metabolic and functional interplay between gut microbiota and fat-soluble vitamins. Crit Rev Food Sci Nutr. 2021;61(19):3211–32. doi: 10.1080/10408398.2020.1793728 32715724

[37] Vangay P, Johnson AJ, Ward TL, Al-Ghalith GA, Shields-Cutler RR, Hillmann BM, et al. US Immigration westernizes the human gut microbiome. Cell. 2018;175(4):962–972.e10. doi: 10.1016/j.cell.2018.10.029 30388453 PMC6498444

[38] O’Keefe SJD, Li JV, Lahti L, Ou J, Carbonero F, Mohammed K, et al. Fat, fibre and cancer risk in African Americans and rural Africans. Nat Commun. 2015;6:6342. doi: 10.1038/ncomms7342 25919227 PMC4415091

[39] Seo D-Y, Heo J-W, Ko JR, Kwak H-B. Exercise and neuroinflammation in Health and Disease. Int Neurourol J. 2019;23(Suppl 2):S82–92. doi: 10.5213/inj.1938214.107 31795607 PMC6905205

[40] Adams C, Conigrave JH, Lewohl J, Haber P, Morley KC. Alcohol use disorder and circulating cytokines: a systematic review and meta-analysis. Brain Behav Immun. 2020;89:501–12. doi: 10.1016/j.bbi.2020.08.002 32805393

[41] Caliri AW, Tommasi S, Besaratinia A. Relationships among smoking, oxidative stress, inflammation, macromolecular damage, and cancer. Mutat Res Rev Mutat Res. 2021;787:108365. doi: 10.1016/j.mrrev.2021.108365 34083039 PMC8287787

[42] Wu H, Ballantyne CM. Metabolic inflammation and insulin resistance in obesity. Circ Res. 2020;126(11):1549–64. doi: 10.1161/CIRCRESAHA.119.315896 32437299 PMC7250139

